# ALDOA Promotes Glycolysis and NLRP3/GSDMD Pyroptosis to Accelerate ALS Progression

**DOI:** 10.1002/acn3.70372

**Published:** 2026-03-24

**Authors:** Kaixin Yan, Yan Jiang, Yuxuan Yong, Tianshuo Zhang, Niannian Zhang, Qianqian Zeng, Xue Gong, Li Meng, Fangfang Bi, Yongmin Liu

**Affiliations:** ^1^ Department of Neurology The Second Affiliated Hospital of Xinjiang Medical University Urumqi China; ^2^ Department of Neurology Fifth Affiliated Hospital of Sun Yat‐Sen University Zhuhai China; ^3^ Department of Neurology Xiangya Hospital Central South University Changsha China

**Keywords:** ALDOA, Aldometanib, ALS, glycolysis, pyroptosis

## Abstract

**Objective:**

Amyotrophic lateral sclerosis (ALS) is characterized by progressive motor neuron degeneration. Glycolytic dysregulation is implicated in disease progression, yet the underlying mechanisms remain unclear. This study investigates how Aldolase A (ALDOA) drives ALS progression through glycolysis‐mediated motor neuron pyroptosis.

**Methods:**

In vivo, tamoxifen‐induced TDP‐43 cKO mice were assessed for motor function (rotarod/suspension tests), motor cortex L‐lactic acid, and ALDOA/NLRP3/GSDMD expression. The ALDOA inhibitor Aldometanib was administered. In vitro, TDP‐43 KO NSC34 cells were used to measure viability, glucose uptake, and L‐lactic acid.

**Results:**

ALS model mice exhibited significant motor deficits, progressive weight loss, and reduced survival. Their motor cortex showed elevated ALDOA expression, L‐lactic acid accumulation, and NLRP3/GSDMD inflammasome activation. Aldometanib treatment suppressed glycolysis, prolonged survival, and slowed disease progression by inhibiting NLRP3/GSDMD‐mediated pyroptosis. In vitro, TDP‐43‐deficient NSC34 cells displayed increased ALDOA levels, enhanced glycolytic flux, NLRP3/GSDMD pathway activation, and impaired proliferation.

**Conclusion:**

We show that ALDOA‐mediated glycolytic dysregulation activates the NLRP3/GSDMD inflammasome, leading to pyroptosis in motor neurons. Pharmacological inhibition of ALDOA alleviates glycolytic dysregulation and extends survival, identifying ALDOA as a potential therapeutic target.

Amyotrophic lateral sclerosis (ALS) is a fatal neurodegenerative disease, primarily influencing upper and lower motor neurons. Its high disability and mortality rates impose a substantial burden on patients' families and society [1]. Recent studies indicate that metabolic dysfunction has become a critical pathogenic mechanism in ALS [2]. Investigations have revealed abnormal glucose metabolism in multiple brain regions of patients carrying C9orf72 repeat expansions [3]. Similarly, fibroblasts with SOD1 mutations exhibit reduced mitochondrial respiration and increased glycolytic flux [4]. These findings collectively highlight that dysregulation of glucose metabolism is a significant hallmark of the disease.

TAR DNA‐binding protein 43 (TDP‐43) is a central pathogenic factor in ALS. Its mutations lead to loss of nuclear function and pathological cytoplasmic aggregation [5]. Beyond disrupting RNA metabolism and causing widespread RNA dysregulation [6], deficiency of TDP‐43 also impairs the DNA damage response [7] and disrupts autophagy‐dependent protein homeostasis [8]. Currently, the pathogenic role of TDP‐43 nuclear depletion in ALS remains unclear, which severely limits clinical treatment for the disease.

Glycolysis, the core energy‐producing pathway that converts glucose to pyruvate/lactate, is essential for cellular function [9, 10]. Recently, abnormal glycolysis phenomena have been observed in Alzheimer's disease (AD) and Parkinson's disease [11, 12]. Intervention in glycolysis process can exert neuroprotective effects and improve the progression of Parkinson's diseases [12]. Aldolase A (ALDOA), as a monitor of glycolysis, is a key enzyme in canonical glycolysis and gluconeogenesis [9]. Our previous research has shown that TDP‐43 M337V mutation enhances its binding to ALDOA and increases its expression, thereby linking TDP‐43 protein disease with glycolysis imbalance [13].

Elevated ALDOA activity in macrophages enhances glycolysis and suppresses AMPK, leading to NLRP3 inflammasome activation, which promotes inflammatory responses and cell death [14]. Pyroptosis, also known as inflammatory necrosis, is a form of programmed cell death [15]. Research indicates that NLRP3 inflammasome‐mediated pyroptosis can exacerbate neuroinflammation and accelerate motor neuron degeneration in ALS [16, 17]. Although studies have established that glycolytic dysfunction can induce neuronal degeneration [18] and that ALDOA can activate NLRP3 [14], it remains to be elucidated whether, in ALS, ALDOA‐mediated glycolytic dysfunction triggers pyroptosis via NLRP3 to directly drive motor neuron degeneration.

Therefore, this study established an ALS mouse model with conditional TDP‐43 knockout (cKO) and a TDP‐43 knockout (KO) cell model. Our results revealed the presence of ALDOA‐dependent glycolysis enhancement in the TDP‐43 deficient ALS model. ALDOA‐mediated aberrant enhancement of glycolysis drives NLRP3/GSDMD pathway activation, ultimately resulting in neuronal pyroptosis, thereby promoting motor neuron degeneration and disease progression in TDP‐43‐deficient ALS models.

## 1 Materials and Methods

### 1.1 Materials

Aldometanib (MCE, HY‐148189, Shanghai, China) was dissolved at a concentration of 1 mg/mL in a solvent consisting of 2% DMSO, 40% PEG 300, 5% Tween‐80, and 53% saline and was freshly prepared prior to use. Fetal bovine serum (FBS) (Lonsera Biotechnology, S711‐001S, Suzhou, China), cell counting kit‐8 (CCK‐8) (Beyotime, C0048M, Shanghai, China), JC‐1 mitochondrial dye (MCE, HY‐15534, Shanghai, China), and tamoxifen (Sigma, T5648, Burlington, USA) were commercially obtained.

### 1.2 Establishment of a Mouse Model

This study utilized a total of 18 C57BL/6 mice (age, 10–16‐week‐old; no specific sex ratio was set due to the use of conditional knockout gene mice) carrying *TDP‐43* ^*flox/WT*^ and *Map2‐Cre* ^*ERT2*^ alleles (Southern Model Organisms) that were housed in a pathogen‐free barrier facility (25°C, 60% humidity, 12‐h light/dark cycle) with ad libitum food/water. Following two generations of breeding, F3 *TDP‐43* ^*flox/flox*^; *Map2‐Cre* ^*ERT2*^ double‐homozygous mice received intraperitoneal injections of tamoxifen (75 mg/kg/day) for five consecutive days at 8 weeks of age to induce neuron‐specific TDP‐43 knockout (designated as TDP‐43 cKO mice). Seven days after tamoxifen induction, the mice were completely randomized using a computer‐generated random number table into three groups (*n* = 6 per group): Control + solvent, TDP‐43 cKO + solvent, and TDP‐43 cKO + Aldometanib. To minimize experimental bias, all behavioral tests and outcome assessments were conducted under single‐blind conditions, where experimenters were unaware of the group assignments. Specifically, blinding was applied during the execution of all behavioral tests (rotarod, suspension), tissue collection, histological evaluation, and quantitative data analysis. The group coding was not revealed until after data analysis was completed. Starting 1 week post‐induction, either the solvent (DMSO + PEG 300 + Tween‐80 + saline) or Aldometanib (5 mg/kg) was administered via oral gavage four times per week until the experimental endpoint. The pharmacological regimen commenced at 8 weeks of age (1 week post‐tamoxifen induction) and was maintained for 4 weeks. The selection of the 5 mg/kg dose was based on literature precedent: prior evidence indicated that a 2 mg/kg dose was insufficient to activate autophagy in the brain [19], whereas a 5 mg/kg dose has been effectively employed in other models [20]; therefore, the higher dose was adopted for our experimental validation. All animal experimental protocols were approved and supervised by the Animal Ethics Committee of The Fifth Affiliated Hospital of Sun Yat‐sen University.

### 1.3 Rotarod Test

Motor function was assessed using a rotarod system. The apparatus was programmed to accelerate linearly from 4 to 40 rpm over a 300‐s period [21]. Prior to formal testing, all mice underwent a 7‐day acclimation training protocol at 49 days of age, following the same acceleration procedure. Only mice capable of consistently remaining on the rod for the full 300 s during training were included in the subsequent study. Formal testing was initiated 1 week after tamoxifen induction and conducted three times per week. During each test session, every mouse performed three consecutive trials with a 30‐min rest interval between trials. The fall latency was recorded to evaluate motor coordination. All behavioral tests were performed at the same time each day to control for potential circadian influences.

### 1.4 Hanging Wire Test

Acclimation training began at 49 days of age. Mice were placed on a wire grid, which was then gently tapped and inverted at 15° per second to a fully inverted position. After 7 days of training, only individuals capable of maintaining grip for 90 s were included in the study. Formal testing commenced 1 week post‐tamoxifen injection, with three sessions per week. During each test, qualified mice were placed on the grid, which was then inverted while timing their hang duration (from full inversion until any limb release), with a 90‐s cut‐off.

### 1.5 Tissue Preparation

Following completion of behavioral assessments, mice were anesthetized. A subset was transcardially perfused and fixed with 4% paraformaldehyde. The brain and lumbar enlargement of the spinal cord were dissected for Nissl and hematoxylin and eosin (H&E) staining, while a portion of the brain tissue was processed for immunofluorescence staining. The remaining mice were perfused, immediately decapitated, and their brains were snap‐frozen in liquid nitrogen and stored at −80°C for subsequent analyses.

### 1.6 Cell Culture

NSC34 cells (Kanglang Biotechnology) were cultured in a Dulbecco's modified Eagle's medium (DMEM; GIBCO, New York, NY, USA) supplemented with 10% FBS and 1% Penicillin–streptomycin at the Fifth Affiliated Hospital of Sun Yat‐sen University. Cryopreserved cells were thawed rapidly in a 37°C water bath, resuspended in a complete medium, centrifuged, and transferred into a fresh culture medium. Cultures were maintained at 37°C in a humidified atmosphere, containing 5% CO_2_. Cells beyond Passage 20 were excluded from experiments.

### 1.7 Cell Infection

NSC34 cells were seeded at a density of 50% and cultured overnight at 37°C. PSLenti‐U6‐shRNA(*Tardbp*)‐CMV‐EGFP‐F2A‐Puro‐WPRE lentiviral dilution or control dilution was prepared and added to the cell culture dishes. This lentiviral construct mediates transient TDP‐43 knockdown via short hairpin RNA (shRNA) targeting the *Tardbp* gene. After 12 h of incubation, the medium was replaced. Cells were then harvested for subsequent experiments 48–72 h post‐transduction to assess the effects of transient TDP‐43 knockdown. The expression levels of viral genes or proteins in the cells were detected using appropriate methods. The efficiency of TDP‐43 knockdown in these transiently transduced cells was confirmed by Western blot and quantitative RT‐PCR analyses, as presented in the Figure S2.

### 1.8 CCK‐8 Assay

Cell viability was determined using the CCK‐8 assay. NSC34 cell suspensions were seeded into 96‐well plates at approximately 100 μL per well at a density of 1 × 10^3^ cells per well. Blank, control, and experimental groups were established with 3–5 replicate wells per group. The TDP‐43 cKO + aldometanib group was treated with 1 nM aldometanib for 24 h. Subsequently, culture medium containing 10% CCK8 reagent was added to each well, followed by incubation at 37°C for 1–4 h. Absorbance was measured at 450 nm. Cell viability was calculated as a percentage relative to the control group after subtracting the average absorbance of blank wells.

### 1.9 Flow Cytometry Detection

The culture medium was removed, and the cells were detached using trypsin. After centrifugation and supernatant removal, cells were twice washed with phosphate‐buffered saline (PBS). Cells were stained with 2‐NBDG (MCE, Shanghai, China) or PI staining solution (Beyotime) for 5–60 min at room temperature in darkness. After staining, cells were washed once with PBS and resuspended at 1 × 10^4^ cells/mL. Flow cytometry was carried out using a Beckman Cytoflex LX instrument.

### 1.10 L‐Lactate Assay

L‐lactate concentrations in cells, cell supernatants, and mouse motor cortex were measured using a colorimetric assay kit (Elabscience, Wuhan, China) according to the manufacturer's instructions. Absorbance was determined at 530 nm using a microplate reader.

### 1.11 Nissl Staining

Brain tissue sections were dewaxed with xylene and rehydrated through graded alcohols. Sections were immersed in Nissl staining solution at 37°C for 5 min, rinsed with distilled water, and mounted with xylene‐based mounting medium to assess neuronal damage. Stained sections were imaged using a high‐resolution digital Pathology slide scanner. Nissl‐positive cells were quantified using GraphPad Prism 10.4.1 software (GraphPad Software Inc., San Diego, CA, USA). Representative images from three independent experiments are illustrated (scale bar: left 20 μm, enlarged view: 50 μm).

### 1.12 Quantitative Reverse TranscriPtion Polymerase Chain Reaction (RT‐qPCR)

Total RNA was extracted using TRIzol reagent (Beyotime). Complementary DNA (cDNA) was synthesized using the HiScript III RT SuperMix for qPCR (Vazyme, Nanjing, China). Target gene amplification was undertaken using the ChamQ Universal SYBR qPCR Master Mix (Vazyme) on an Supplementary ABI QuantStudio 7 Flex system. Relative gene exPression levels were calculated using the 2^−ΔΔC*t*^ method, with β‐actin serving as the internal reference gene for normalization. Primer sequences are listed in Table S1.

### 1.13 Enzyme‐Linked Immunosorbent Assay (ELISA)

IL‐1β concentrations in cell supernatants and mouse motor cortex were measured using an ELISA kit (Elabscience) per manufacturer's instructions. Absorbance was recorded at 450 nm.

### 1.14 Immunofluorescence Staining

Frozen brain sections were thrice washed with PBS and blocked for 1 h at room temperature. Sections were incubated overnight at 4°C with the following primary antibodies: anti‐ALDOA (1:200, ab252953, Abcam, Cambridge, UK), anti‐TDP‐43 (1:200, 10,782–2‐AP, Proteintech, Rosemont, IL, USA), anti‐NLRP3 (1:200, 30,109–1‐AP, Proteintech), and anti‐N‐GSDMD (1:100, ab215203, Abcam). After washing, sections were incubated with horseradish peroxidase (HRP)‐conjugated secondary antibodies for 2 h at room temperature in darkness. Following PBS washing, sections were treated with TSA dye solution (G1256, Servicebio, Wuhan, China) for 15 min at room temperature. Tissue sections were incubated overnight at 4°C with anti‐NeuN antibody (1:200, ET1602‐12, HUABIO), followed by washing and incubation with appropriate secondary antibodies for 2 h at room temperature. Sections were thereafter mounted using a DAPI‐containing mounting medium. Images were acquired using a digital slide scanner. Representative images from three independent experiments are illustrated (scale bars: left panel, 20 μm; enlarged view, 200 μm).

### 1.15 Western Blotting

Total Protein was extracted from motor cortex and NSC34 cells using ice‐cold RIPA buffer supplemented with protease/phosphatase inhibitors. Protein concentration was quantified using a BCA kit (Solarbio, Beijing, China). Equal amounts of protein (10–30 μg) were separated on 6%–12% sodium dodecyl sulfate–Polyacrylamide gel electrophoresis (SDS‐PAGE) gels and transferred onto polyvinylidene difluoride (PVDF) membranes. Membranes were blocked with 5% skim milk in TBST and incubated overnight at 4°C with the following primary antibodies: anti‐ALDOA (1:1000, 11,217–1‐AP, Proteintech), anti‐TDP‐43 (1:1000, 10,782–2‐AP, Proteintech), anti‐IL‐1β (1:800, 16,806–1‐AP, Proteintech), anti‐NLRP3 (1:1500, 30,109–1‐AP, Proteintech), anti‐Caspase‐1 (1:1000, 20,770–1‐AP, Proteintech), anti‐GSDMD (1:1000, 22,915–1‐AP, Proteintech), and anti‐Tubulin (1:5000, 11,224–1‐AP, Proteintech). Following TBST washing, membranes were incubated with secondary antibodies (1:5000, Jackson ImmunoResearch, West Grove, PA, USA) for 1 h at room temperature and developed using ECL plus. Tubulin served as a loading control. The band intensity of each target protein was quantified using ImageJ software and normalized to the corresponding Tubulin signal. The relative protein expression level was expressed as the ratio of target protein intensity to Tubulin intensity. Band intensities were analyzed with ImageJ software.

### 1.16 Statistical Analysis

All data were collected from at least three independent experiments and were presented as mean ± standard deviation (SD). Statistical analysis was performed using GraphPad Prism 10.4.1 software. Comparisons among multiple groups were performed using one‐way analysis of variance (ANOVA), behavioral data were analyzed using two‐way ANOVA, and pairwise comparisons between two groups were conducted using unpaired *t*‐test. *p* below 0.05 was considered statistically significant.

## 2 Results

### 2.1 Successful Generation of TDP‐43 cKO Mice With ALS‐Like Phenotypes

After crossing *Map2‐Cre* ^*ERT2*^ mice with *TDP‐43* ^*flox/WT*^ mice for two generations, F3 offspring at 4 weeks of age underwent tail genotyping to obtain double‐positive homozygous mice for both *TDP‐43* ^*flox/flox*^ and *Map2‐Cre* ^*ERT2*^. At 8 weeks of age, the mice received intraperitoneal injections of tamoxifen. Western blot and RT‐qPCR analyses were used to validate the knockout efficiency of the TDP‐43 gene. The results demonstrated that TDP‐43 mRNA and protein levels in the motor cortex of *TDP‐43* ^*flox/flox*^; *Map2‐Cre* ^*ERT2*^ mice were significantly downregulated compared with *TDP‐43* ^*flox/flox*^ mice. Immunofluorescence staining for Map2 and TDP‐43 revealed that after tamoxifen induction, TDP‐43 was specifically knocked out in the motor cortex neurons of *TDP‐43* ^*flox/flox*^; *Map2‐Cre* ^*ERT2*^ mice compared with the control group. The breeding strategy and validation for *TDP‐43* ^*flox/flox*^; *Map2‐Cre* ^*ERT2*^ mice are detailed in Figure S1. Therefore, this mouse model was referred to as the TDP‐43 cKO mouse in the present study.

**FIGURE 1:**
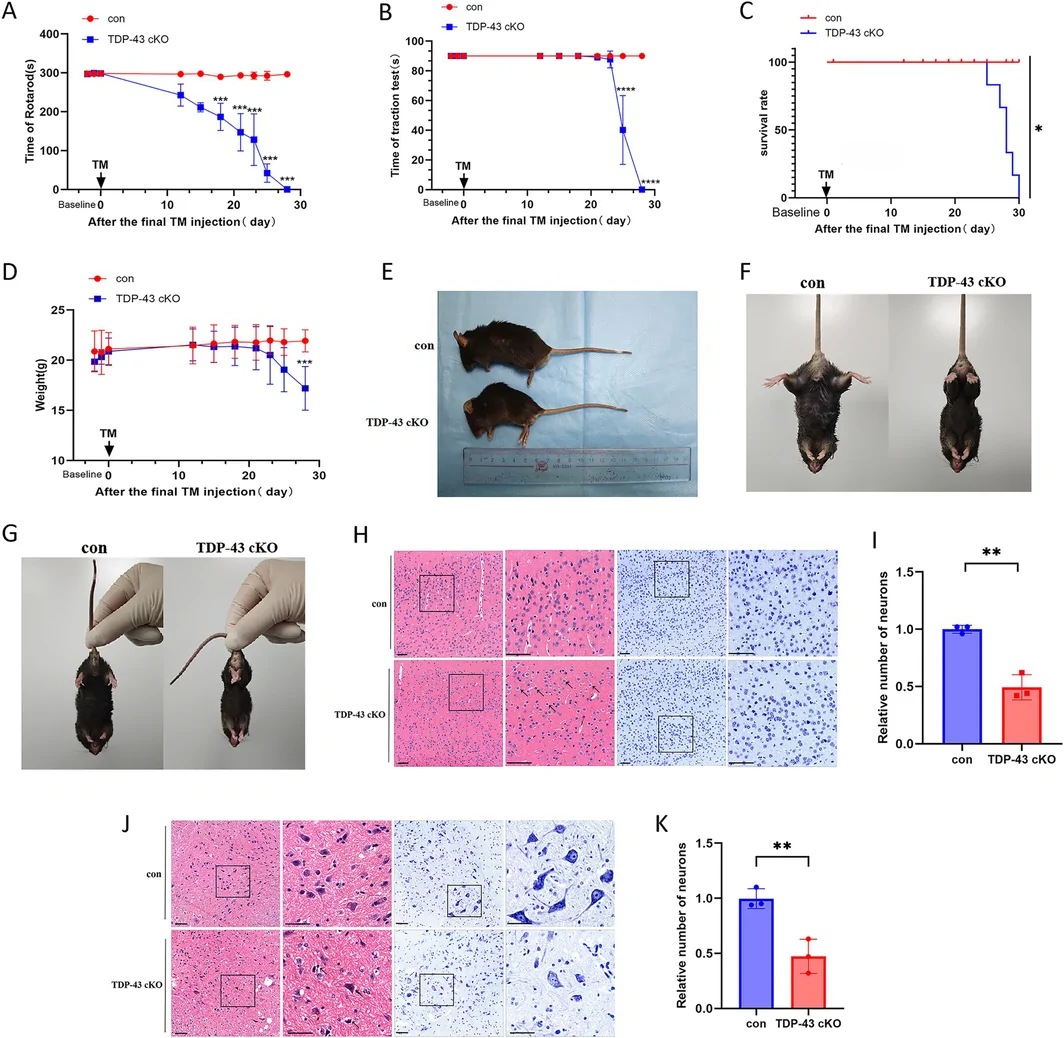
Successful generation of TDP‐43 cKO mice exhibiting ALS‐like phenotypes. (A, B) Statistical graphs showing results of the rotarod fatigue test and wire hang test, respectively, for each group of mice. (C) Survival analysis graph for each group of mice. (D) Statistical graph of body weight changes for each group of mice. (E) Morphology comparison of mice at the moribund stage for each group. (F) Comparison of hindlimb extension for each group of mice. (G) Comparison of flaccid tails for each group of mice. (H) Representative images of H&E staining (left panel) and Nissl staining (right panel) in the motor cortex of control and TDP‐43 cKO mice. (I) Statistical graph of Nissl‐positive neurons. (J) Representative images of H&E staining (left panel) and Nissl staining (right panel) in the ventral horn of the lumbar spinal cord enlargement of control and TDP‐43 cKO mice. (K) Statistical graph of Nissl‐positive neurons Scale bars: 20 μm (H left, J left); 50 μm for enlarged views below (H right, J right). Data are presented as mean ± standard deviation (Mean ± SD). (I, K): Unpaired *t*‐test. (A–D): Two‐way analysis of variance (two‐way ANOVA). Compared with the control (con) group: *p < 0.05, **p < 0.01, ***p < 0.001.

Behavioral test results indicated that starting from day 12 Post‐tamoxifen induction, TDP‐43 cKO mice exhibited a downward trend in rotarod performance compared with the control group, which significantly reduced by day 18 (Figure 1A, *p* < 0.0001). Hanging wire test results revealed that compared with the control group, TDP‐43 cKO mice demonstrated significantly reduced forelimb hanging ability by day 24 Post‐tamoxifen induction, and they also exhibited comPlete Paralysis and an inability to hang on the wire grid around day 28 (Figure 1B, *p* < 0.0001). By day 28 Post‐tamoxifen induction, TDP‐43 cKO mice approached a moribund state, exhibiting significantly shorter survival time than control mice (Figure 1C, *p* = 0.0008). At day 28 Post‐tamoxifen induction, compared with the control group, TDP‐43 cKO mice exhibited reduced body weight (Figure 1D, *p* = 0.0008), smaller body size (Figure 1E), significant atrophy of muscles throughout the body, Particularly in the hindlimbs (Figure 1F), and flaccid tails (Figure 1G). These results demonstrated that TDP‐43 cKO mice develoPed ALS‐like behavioral phenotypes, including reduced motor ability, muscle atrophy, Paralysis, and death, following tamoxifen‐induced TDP‐43 knockout.

H&E staining of the motor cortex at 28 days post‐tamoxifen induction revealed neuronal atrophy in TDP‐43 cKO mice (Figure 1H). Nissl staining indicated a significant reduction in neuronal count in the motor cortex of TDP‐43 cKO mice compared with the control group (Figure 1H,I, *p* = 0.0021). Similarly, H&E staining (Figure 1J) and Nissl staining of the ventral horn in the lumbar spinal cord enlargement at 28 days post‐induction also revealed a significantly decreased number of neurons in TDP‐43 cKO mice versus controls (Figure 1J,K, *p* = 0.0072). These findings indicated that following tamoxifen‐induced TDP‐43 knockout, TDP‐43 cKO mice developed ALS‐like pathological phenotypes in both the motor cortex and spinal cord ventral horn, characterized by motor neuron degeneration and loss. These results confirmed the successful establishment of the mouse model of ALS. According to behavioral outcomes, post‐induction days 12–14 were defined as the early symptomatic stage of ALS, day 21 as the mid‐stage, and day 28 as the terminal stage.

### 2.2 Upregulation of ALDOA Expression Level and Increased L‐Lactate Level in the Motor Cortex of TDP‐43 cKO Mice

**FIGURE 2:**
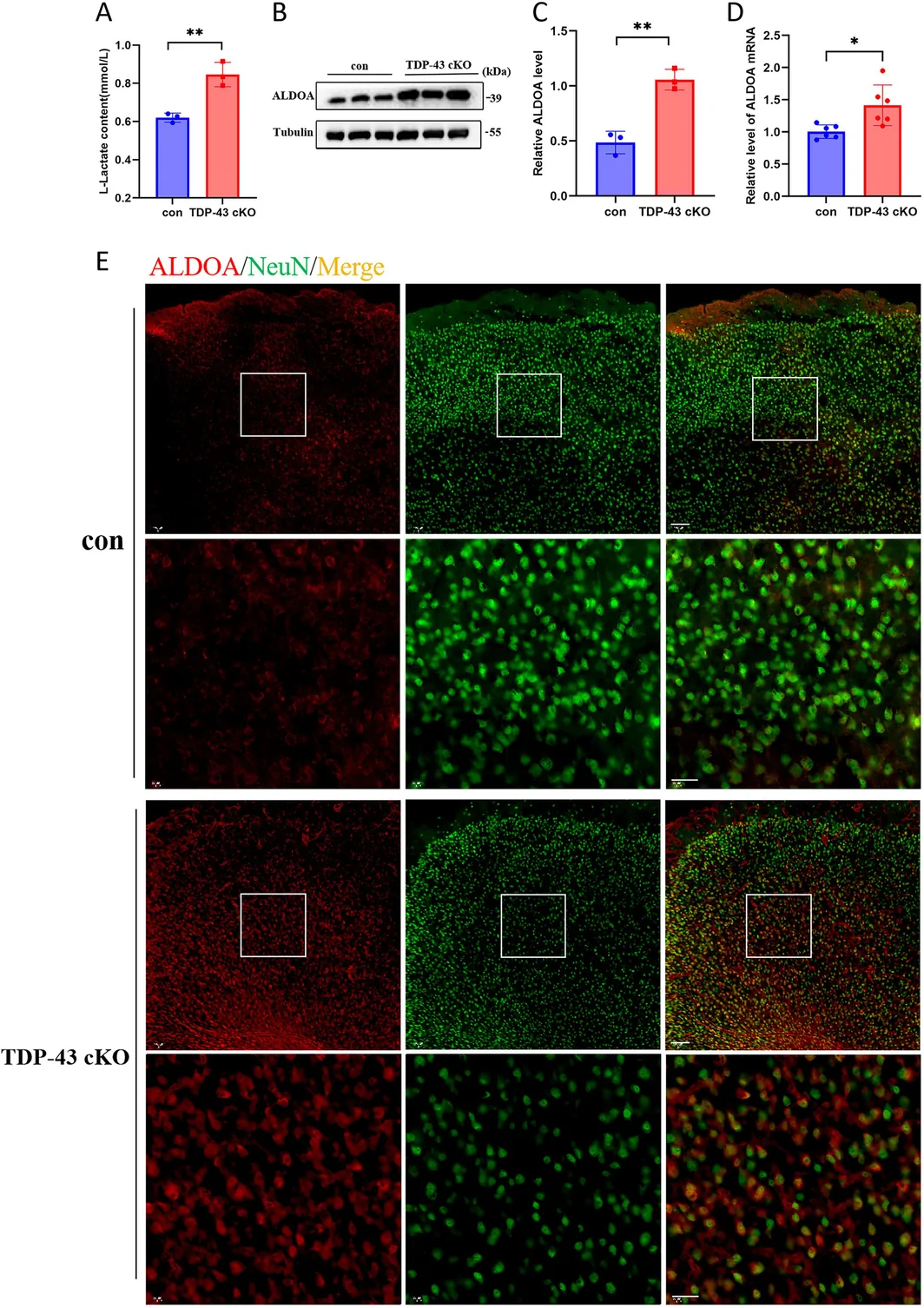
Increased lactate levels and elevated ALDOA expression in the motor cortex of TDP‐43 cKO mice. (A) Statistical graph of L‐lactate levels in the motor cortex across mouse groups. (B) Representative Western blot images of ALDOA in the motor cortex across mouse groups. (C) Statistical graph of ALDOA protein expression in the mouse motor cortex. (D) Statistical graph of ALDOA mRNA levels in the motor cortex across mouse groups detected by RT‐qPCR. (E) Representative immunofluorescence colocalization images of ALDOA and NeuN in the motor cortex across mouse groups. Scale bar: 100 μm (upper panels); 20 μm for enlarged views (lower panels). Data are presented as mean ± standard deviation (Mean ± SD). (A, C and D): Unpaired *t*‐test. Compared with the control (con) group: *p < 0.05, **p < 0.01.

L‐lactate is the end product of anaerobic glycolysis, and changes in its level can indirectly reflect the extent of anaerobic glycolysis in tissues. Measurement of L‐lactate level in the motor cortex across mouse groups revealed that, compared with the control group, TDP‐43 cKO mice exhibited significantly elevated L‐lactate level in the motor cortex during the terminal stage of ALS disease (Figure 2A, *p* = 0.0046). This indicates L‐lactate accumulation in the motor cortex of ALS mice, reflecting that the anaerobic glycolytic process may be enhanced. The expression levels of the key glycolytic enzyme ALDOA was detected in the motor cortex. The results revealed that, compared with the control group, TDP‐43 cKO mice exhibited significantly increased expression levels of ALDOA mRNA (Figure 2D, *p* = 0.0128) and protein (Figure 2B,C, *p* = 0.0021) in the motor cortex during the terminal stage of ALS. Immunofluorescence assay demonstrated clear colocalization of ALDOA with neurons in the motor cortex of TDP‐43 cKO mice (Figure 2E). These findings indicated the elevated ALDOA expression level and enhanced anaerobic glycolysis in the motor cortex of ALS mice.

### 2.3 TDP‐43 Knockdown in NSC34 Cells Could Lead to Enhanced Anaerobic Glycolysis and Reduced Cell Viability

**FIGURE 3:**
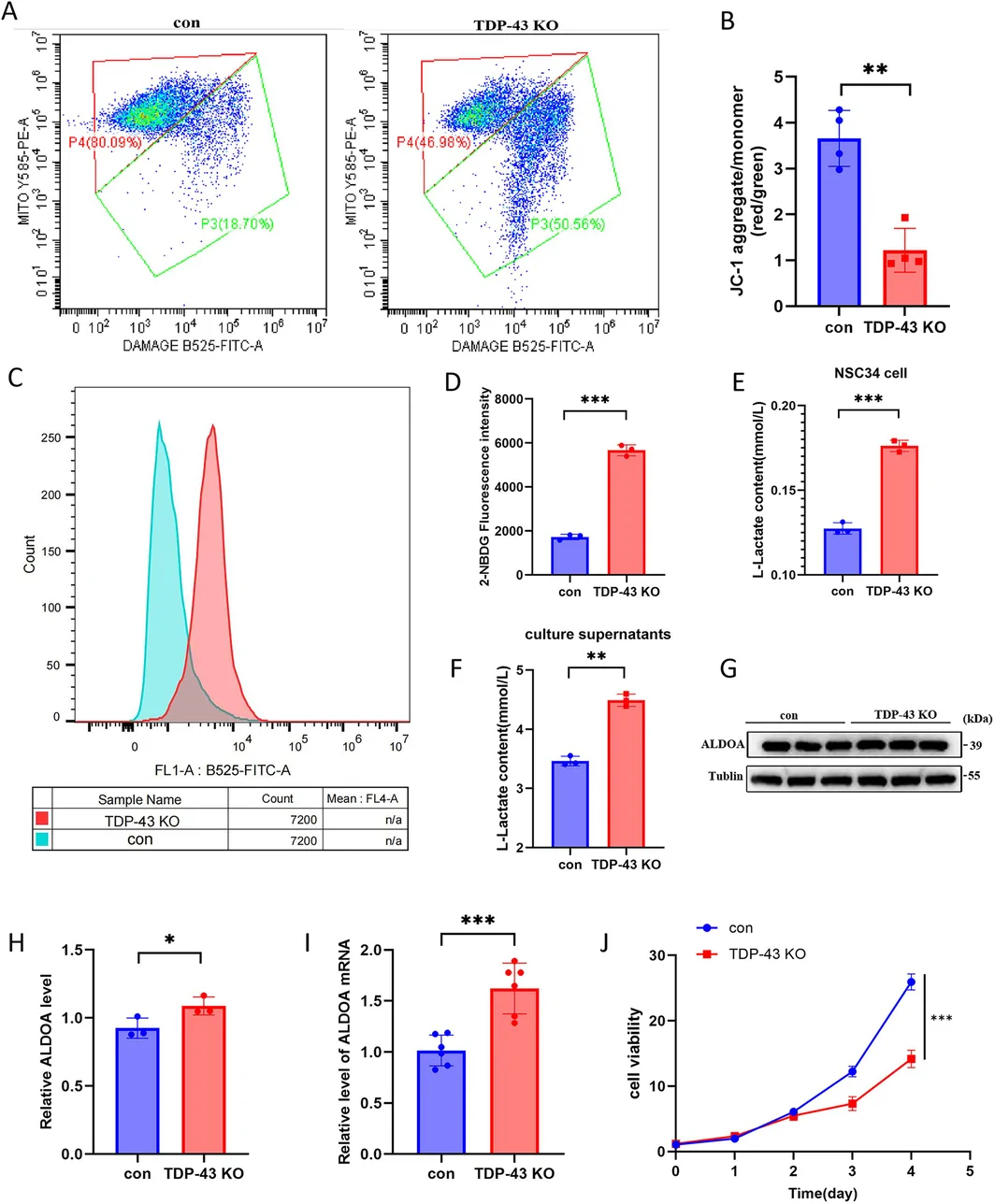
TDP‐43 knockdown in NSC34 cells induces enhanced anaerobic glycolysis and reduced cell viability. (A, B) Representative flow cytometry plots (A) and quantification graph (B) of JC‐1 fluorescence changes across cell groups. (C, D) Representative flow cytometry plots (C) and quantification graph (D) of 2‐NBDG‐labeled cellular glucose uptake rates. (E, F) Quantification graphs of L‐lactate levels in intracellular compartments (E) and culture supernatants (F) across groups. (G, H) Representative Western blot images (G) and quantification graph (H) of ALDOA expression across cell groups. (I) Quantification graph of ALDOA mRNA levels across cell groups. (J) Cell viability results across groups detected by CCK‐8 assay. Data are presented as mean ± standard deviation (Mean ± SD). (B, D–F, H and I): Unpaired *t*‐test. (J): Two‐way analysis of variance (two‐way ANOVA). Compared with the control (con) group: *p < 0.05, **p < 0.01, ***p < 0.001.

Following 72 h lentiviral infection with short hairpin RNA (shRNA), NSC34 motor neuron cells in the TDP‐43 KO group exhibited significantly reduced TDP‐43 mRNA and protein expression compared with the control group, confirming the successful establishment of the TDP‐43 knockdown cellular model (Figure S2). Flow cytometry analysis of JC‐1 changes (reflecting mitochondrial membrane potential) revealed that, compared with the control group, the TDP‐43 KO group exhibited decreased red fluorescence and increased green fluorescence in JC‐1 staining. This indicates reduced mitochondrial membrane potential, suggesting mitochondrial impairment in cells following TDP‐43 knockdown (Figure 3A,B, *p* = 0.0009). 2‐NBDG, a fluorescent glucose analog, was utilized to assess cellular glucose uptake as an indicator of glycolytic activity. TDP‐43 KO cells exhibited significantly increased glucose uptake compared with the control group (Figure 3C,D, *p* < 0.0001). The elevated glucose uptake, identified in the context of mitochondrial damage, further suggests the enhanced anaerobic glycolysis. Subsequent measurement of L‐lactate levels in cell and culture supernatants revealed significantly elevated L‐lactate levels in TDP‐43 KO cells both intracellularly (Figure 3E, *p* < 0.0001) and in the supernatant (Figure 3F, *p* = 0.0002), indicating intensified anaerobic glycolysis in TDP‐43‐knockdown NSC34 cells. ALDOA expression levels were further assessed, revealing a significant increase in both ALDOA mRNA (Figure 3I, *p* = 0.0005) and protein expression (Figure 3G,H, *p* = 0.0484) in the TDP‐43 KO group compared with the control group. These findings indicated that TDP‐43 knockdown enhanced anaerobic glycolysis in NSC34 cells. Cell viability, measured using the CCK‐8 assay, was significantly reduced in TDP‐43 KO cells relative to controls (Figure 3J, *p* < 0.0001), suggesting that TDP‐43 deficiency could impair the viability of NSC34 cells. Collectively, the results demonstrated that TDP‐43 knockdown could induce mitochondrial dysfunction, promote anaerobic glycolysis, and reduce cell viability in motor neuron‐like NSC34 cells.

### 2.4 Targeting ALDOA Inhibited Anaerobic Glycolysis and Prolonged Survival in ALS Mice

**FIGURE 4:**
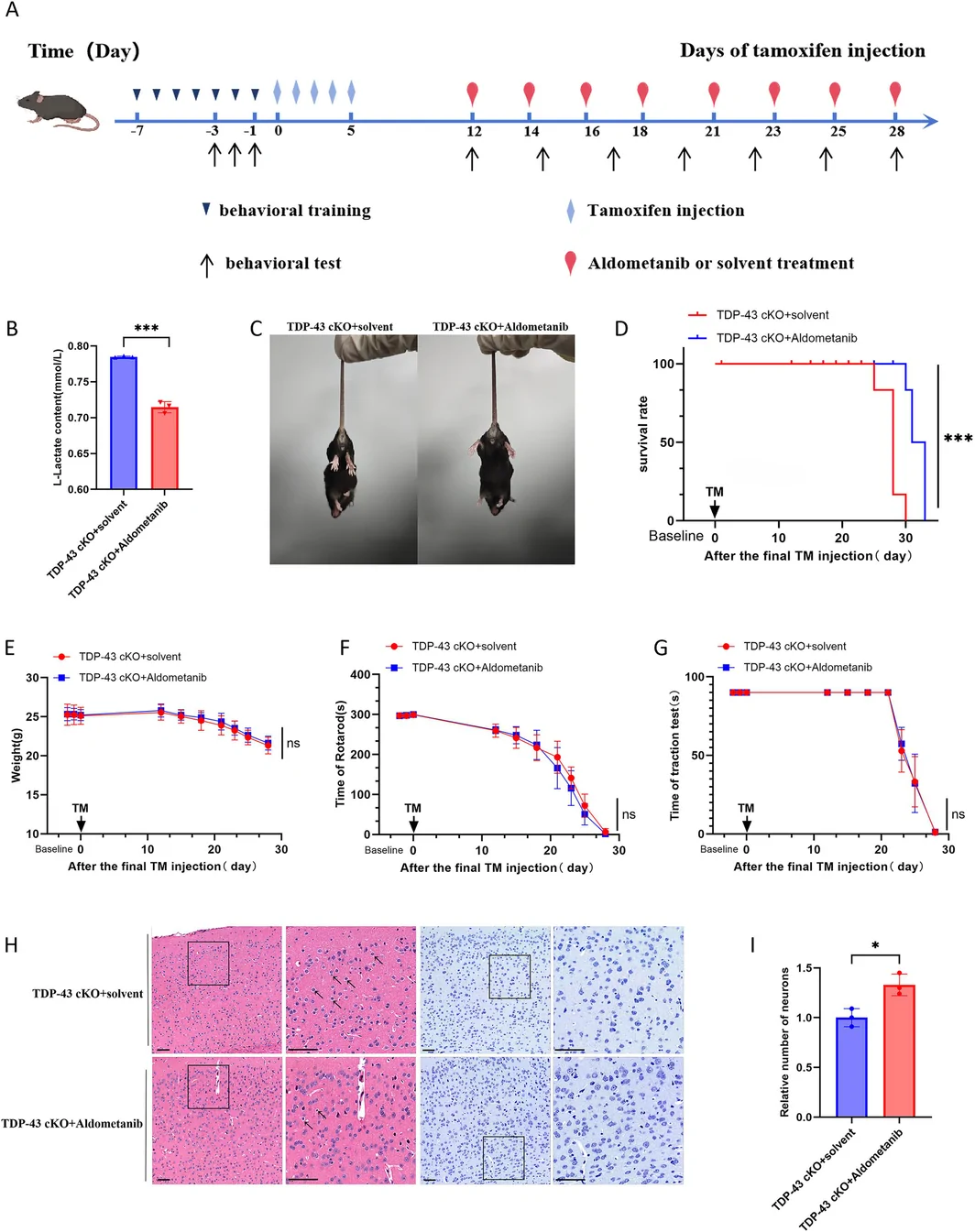
Aldometanib attenuates anaerobic glycolysis and prolongs survival in TDP‐43 cKO mice. (A) Experimental flowchart of drug intervention. (B) Statistical graph of L‐lactate levels in the motor cortex across groups. (C) Morphology comparison of mice across groups at 28 days post‐tamoxifen induction. (D) Survival analysis of mouse groups. (E) Statistical graph of body weight changes across groups. (F, G) Results of rotarod fatigue test (F) and wire hang test (G) across groups. (H) Representative H&E staining (left panel) and Nissl staining (right panel) of the motor cortex in both mouse groups. (I) Statistical graph of Nissl‐positive neurons. Scale bars: 20 μm (H left); 50 μm for enlarged views (H right). Data are presented as mean ± standard deviation (Mean ± SD). (B, I): Unpaired *t*‐test. (D–G): Two‐way analysis of variance (two‐way ANOVA). Compared with the TDP‐43 cKO + solvent group: *p < 0.05, ***p < 0.001.

TDP‐43 cKO mice were administered either solvent or Aldometanib to inhibit ALDOA activity, and the experimental flowchart is illustrated in Figure 4A. The results demonstrated that the TDP‐43 cKO + Aldometanib group exhibited significantly reduced L‐lactate levels in the motor cortex compared with the TDP‐43 cKO + solvent group at day 28 post‐tamoxifen induction (Figure 4B, *p* = 0.0001), indicating decreased lactate production and suppressed anaerobic glycolysis following aldometanib intervention.

At the ALS terminal stage, compared with the TDP‐43 cKO + solvent group, the TDP‐43 cKO + aldometanib group showed attenuated hindlimb muscle atrophy (Figure 4C) and significantly prolonged survival time (Figure 4D, 28 days in TDP‐43 cKO + solvent group vs. 33 days in TDP‐43 cKO + aldometanib group, *p* = 0.0008). No significant differences were identified in body weight changes (Figure 4E, *p* > 0.05) or performance in rotarod and wire hang tests (Figure 4F,G, *p* > 0.05). H&E staining of the motor cortex in terminal‐stage ALS mice (at day 28 post‐tamoxifen induction) revealed attenuated neuronal atrophy in the TDP‐43 cKO + Aldometanib group versus the TDP‐43 cKO + solvent group (Figure 4H). Nissl staining demonstrated a significantly increased neuronal count in the motor cortex in the TDP‐43 cKO + Aldometanib group compared with the solvent group (Figure 4H,I, *p* = 0.0153). These results indicated that Aldometanib could suppress anaerobic glycolysis, mitigate ALS pathological progression, and prolong survival in ALS mice.

### 2.5 Targeting ALDOA Both Enhanced Anaerobic Glycolysis and Cell Death Caused by TDP‐43 Knockdown

**FIGURE 5:**
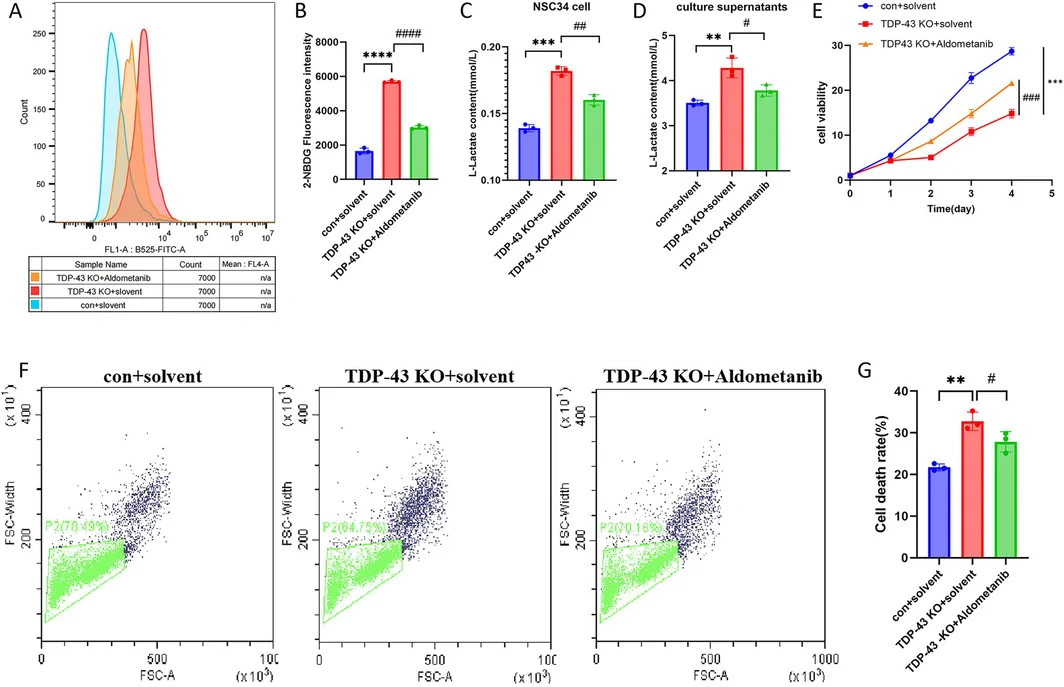
ALDOA inhibition suppresses enhanced anaerobic glycolysis and cell death induced by TDP‐43 knockdown. (A, B) Representative flow cytometry plots (A) and quantification graph (B) of 2‐NBDG‐labeled glucose uptake rates. (C, D) Quantification graphs of L‐lactate levels in intracellular compartments (C) and culture supernatants (D) across groups. (E) Cell viability results across groups treated with 1 nM aldometanib for 24 h detected by CCK‐8 assay. (F) Flow cytometry detection of PI‐labeled cell death. (G) Percentage of dead cells across groups. Data are presented as mean ± standard deviation (Mean ± SD). (B, C, D, G): One‐way analysis of variance (one‐way ANOVA). Comparisons between the con + solvent group and the TDP‐43 KO + solvent group were performed using unpaired *t*‐tests, while comparisons between the TDP‐43 KO + solvent group and the TDP‐43 KO + aldometanib group were conducted using unpaired *t*‐tests. (E): Two‐way analysis of variance (two‐way ANOVA). Compared with the con + solvent group: **p < 0.01, ***p < 0.001, ****p < 0.0001. Compared with the TDP‐43 KO + solvent group: #*p* < 0.05, ##*p* < 0.01, ###*p* < 0.001, ####*p* < 0.0001.

NSC34 cells with TDP‐43 knockdown were treated with 1 nM aldometanib for 24 h. This concentration was selected based on a pilot experiment showing that 1 nM exhibited the optimal effect in suppressing inflammation (Figure S3). Flow cytometry using fluorescently labeled 2‐NBDG revealed significantly reduced glucose uptake in the TDP‐43 KO + aldometanib group compared with the TDP‐43 KO + solvent group (Figure 5A,B; *p* < 0.0001). Measurement of L‐lactate level indicated that after treatment with aldometanib, the levels of L‐lactic acid in cells (Figure 5C; *p* = 0.0005) and culture supernatant (Figure 5D; *p* = 0.015) were significantly reduced. Subsequent assessment of cell viability and death revealed that aldometanib treatment significantly increased cell viability (Figure 5E; *p* < 0.0001) and reduced cell death (Figure 5F,G; *p* = 0.0493) compared with the TDP‐43 KO + solvent group. These findings demonstrated that aldometanib could mitigate both enhanced glycolytic activity and cell death associated with TDP‐43 deficiency in NSC34 cells.

### 2.6 ALDOA Induced Motor Neuron Degeneration and Death in ALS Mice via NLRP3/GSDMD‐Mediated PyroPtosis

**FIGURE 6:**
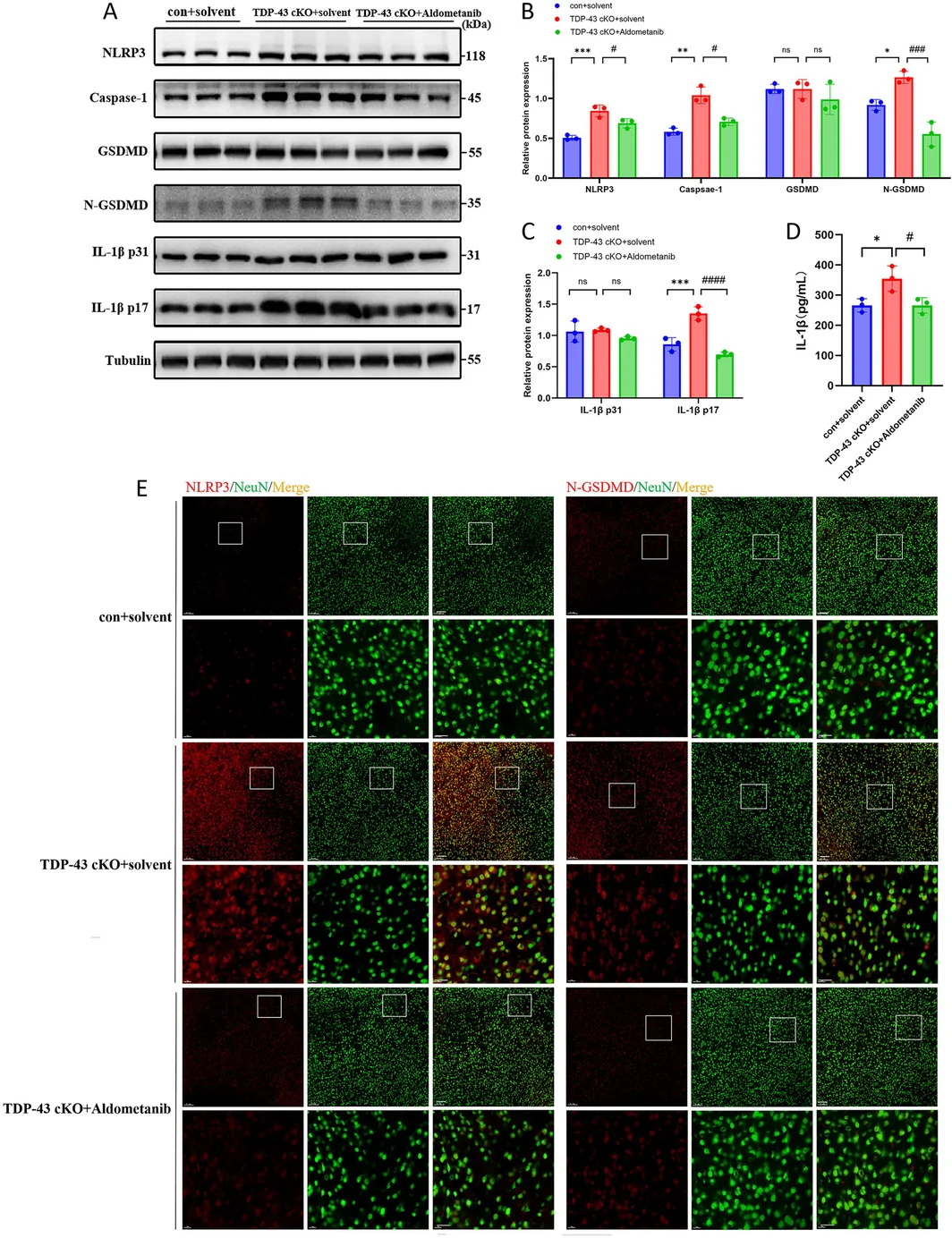
ALDOA Inhibition Attenuates NLRP3/GSDMD‐Mediated pyroptosis. (A) Representative Western blot of NLRP3, Caspase‐1, GSDMD, N‐GSDMD, and IL‐1β in motor cortex. (B, C) Quantification of protein expression from (A). (D) IL‐1β levels in motor cortex (ELISA). (E) NLRP3/NeuN and N‐GSDMD/NeuN immunofluorescence colocalization. Scale bars: 20 μm (upper panels); 200 μm (lower panels). Data = mean ± SD. Statistical tests: (B–D) One‐way ANOVA, Comparisons between the con + solvent group and the TDP‐43 cKO + solvent group were performed using unpaired *t*‐tests, while comparisons between the TDP‐43 cKO + solvent group and the TDP‐43 cKO + aldometanib group were conducted using unpaired *t*‐tests. Compared with the con + solvent group: *p < 0.05, **p < 0.01, ***p < 0.001, Compared with the TDP‐43 cKO + solvent group: #*p* < 0.05, ##*p* < 0.01, ###*p* < 0.001, #### *p* < 0.0001.

This study further explored how ALDOA‐dependent glycolysis abnormalities affect neuronal degeneration and the progression of ALS after the loss of TDP‐43 function. Western blotting results indicated that, compared with the control + solvent group, the expression levels of NLRP3 (*p* = 0.008), Caspase‐1 (*p* = 0.0005), and N‐GSDMD (*p* = 0.0171) were significantly upregulated in the motor cortex in the TDP‐43 cKO + solvent group. Following intervention with aldometanib, the expression levels of NLRP3 (*p* = 0.0341), Caspase‐1 (*p* = 0.0029), and N‐GSDMD (*p* = 0.0004) were significantly downregulated in the motor cortex in the TDP‐43 cKO + aldometanib group compared with the TDP‐43 cKO + solvent group (Figure 6A,B). Immunofluorescence results demonstrated that the NLRP3 and N‐GSDMD were co‐localized with neurons (Figure 6E), indicating that NLRP3/GSDMD‐mediated pyroptosis occurs predominantly in motor neurons and is suppressed by glycolytic inhibition.

ELISA and Western blotting detected IL‐1β level in the motor cortex of the mouse groups. The results revealed that IL‐1β level was significantly elevated in the motor cortex in the TDP‐43 cKO + solvent group compared with the control + solvent group (ELISA:*p* = 0.03; WB:*p* = 0.0014). Following intervention with aldometanib, IL‐1β level was significantly reduced in the motor cortex in the TDP‐43 cKO + aldometanib group compared with the TDP‐43 cKO + solvent control group (Figure 6A,C,D; ELISA: *p* = 0.0297; WB: *p* = 0.0003). These findings demonstrated that aldomidanib inhibits pyroptosis, thereby alleviating the resultant neuroinflammation.

Beyond neuronal pyroptosis, microgliosis was observed in the motor cortex of TDP‐43 cKO mice (Figure S4), suggesting that neuronal metabolic and pyroptotic dysregulation coincides with a glia‐associated inflammatory milieu. Together, these results establish that ALDOA‐driven glycolysis promotes NLRP3/GSDMD‐mediated pyroptosis and neuroinflammation, driving motor neuron degeneration in ALS and highlighting this pathway as a promising therapeutic target.

### 2.7 ALDOA Induced Pyroptosis in TDP‐43 KO NSC34 Cells via NLRP3/GSDMD

**FIGURE 7:**
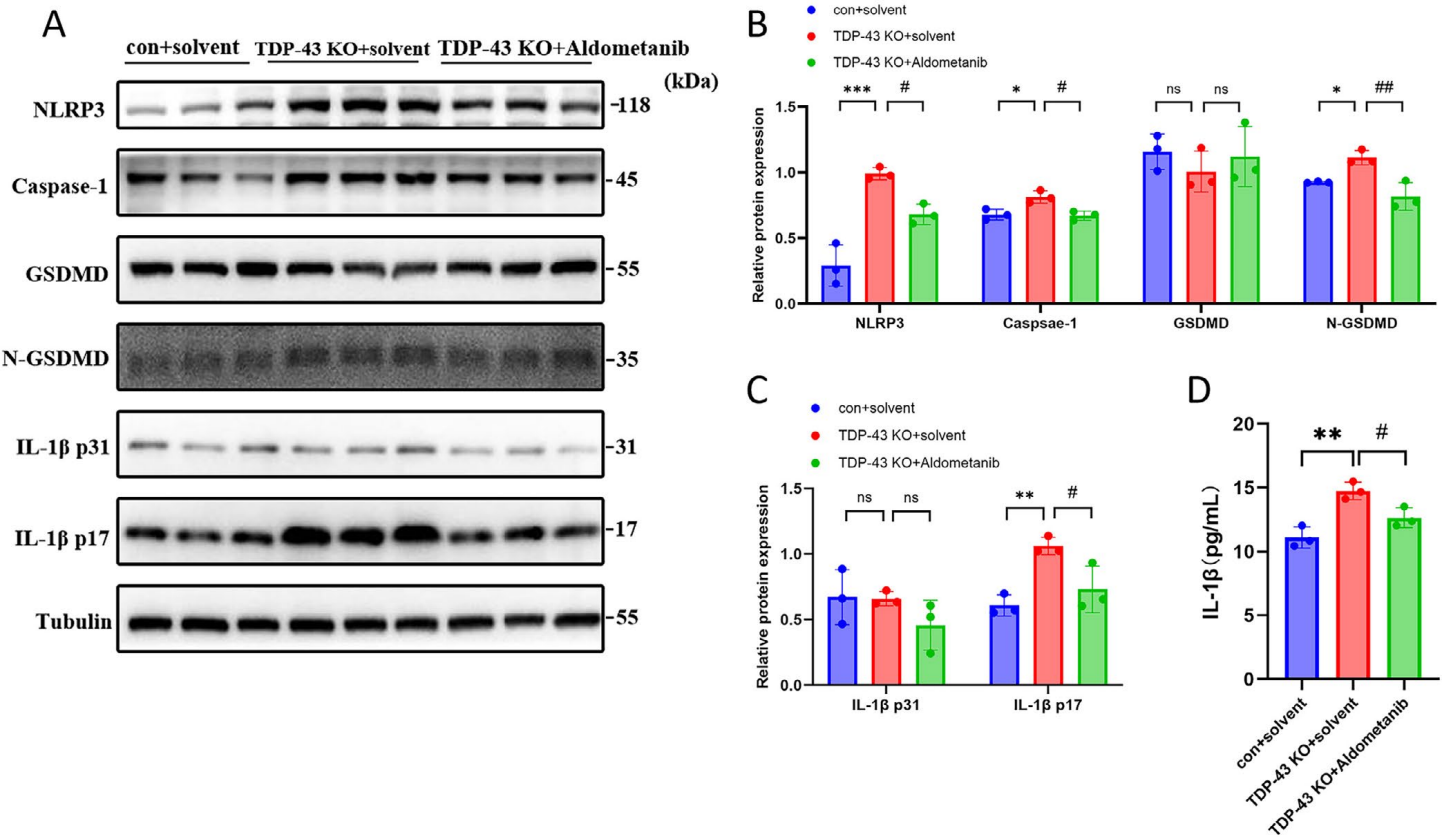
ALDOA Inhibition Attenuates NLRP3/GSDMD‐Mediated pyroptosis in TDP‐43 KO NSC34 Cells. (A) Quantification of NLRP3, Caspase‐1, GSDMD, N‐GSDMD, and IL‐1β protein bands. (B, C) Densitometric analysis of immunoblot bands from (A). (D) IL‐1β levels (ELISA). Data = mean ± SD. Statistical tests: (B–D) One‐way ANOVA. Comparisons between the con + solvent group and the TDP‐43 KO + solvent group were performed using unpaired *t*‐tests, while comparisons between the TDP‐43 KO + solvent group and the TDP‐43 KO + aldometanib group were conducted using unpaired *t*‐tests. Compared with the con + solvent group: *p < 0.05, **p < 0.01, ***p < 0.001. Compared with the TDP‐43 KO + solvent group: #*p* < 0.05, ##*p* < 0.01.

Following 24‐h treatment of TDP‐43 KO NSC34 cells with 1 nM aldometanib, Western blot analysis was performed to evaluate the expression levels of NLRP3, Caspase‐1, GSDMD, N‐GSDMD, and IL‐1β. Compared with the control + solvent group, the TDP‐43 KO + solvent group exhibited significantly increased levels of NLRP3 (*p* = 0.0004), Caspase‐1 (*p* = 0.0186), N‐GSDMD (*p* = 0.0352), and IL‐1β p17 (*p* = 0.008). Treatment with aldometanib significantly reduced the expression levels of these proteins in the TDP‐43 KO + aldometanib group compared with the TDP‐43 KO + solvent group: NLRP3 (*p* = 0.0257), Caspase‐1 (*p* = 0.0143), N‐GSDMD (*p* = 0.0046), and IL‐1β p17 (*p* = 0.0326) (Figure 7A–C).

Consistent with these results, ELISA indicated a significant increase in IL‐1β secretion in the TDP‐43 KO + solvent group relative to the control + solvent group (*p* = 0.0027), which was significantly reduced following aldometanib treatment (*p* = 0.0345) (Figure 7D). These findings in TDP‐43 KO cells align with those noted in the mouse model of ALS. Collectively, the results indicated that TDP‐43 deficiency could promote pyroptosis and neuroinflammation through activation of the NLRP3/GSDMD pathway. Aldometanib could attenuate pyroptosis and neuroinflammation by inhibiting NLRP3 inflammasome activation and GSDMD cleavage.

## 3 Discussion

Notably, ALS involves multifaceted pathogenic mechanisms [22]. In the present study, TDP‐43 cKO mice and TDP‐43‐deficient NSC34 cell models were established to investigate the pathological mechanisms driven by TDP‐43 deficiency. The results demonstrated that neuronal loss of TDP‐43 could upregulate ALDOA‐mediated anaerobic glycolysis, thereby accelerating the progression of ALS. Pharmacological inhibition of ALDOA with aldometanib suppressed glycolytic activity, prolonged the survival time of TDP‐43 mice, and partly rescued neuronal degeneration. Mechanistically, the loss of TDP‐43 function leads to high expression of ALDOA, which activated the NLRP3/GSDMD signaling pathway, and ultimately promoted pyroptosis of motor neurons.

Existing TDP‐43 animal models are generally categorized into two types: overexpression and TDP‐43 deletion models [23]. In TDP‐43 mutant models, the cytoplasmic mislocalization and aggregation of the TDP‐43 protein lead to neurotoxic effects and progressive behavioral impairments such as cognitive decline, motor dysfunction, and eventual paralysis [24]. In a mouse model of spinal motor neuron depletion of TDP‐43 (*TDP‐43* ^*flox/flox*^; *HB9‐Cre* ^*ERT2*^), ALS‐like phenotypes were observed, including spinal kyphosis, motor dysfunction, muscle atrophy, and proliferation of spinal astrocytes [25]. *TDP‐43* ^*flox/flox*^; *ChAT‐IRES‐Cre* ^*ERT2*^‐mediated motor neuron‐specific deletion of TDP‐43 results in motor dysfunction and neuronal loss in mice, with a reported median survival of 44 weeks [26]. Compared with heterozygous knockouts or models with deletion restricted to specific neuronal subtypes or regions, this model (*TDP‐43* ^*flox/flox*^; *Map2‐Cre* ^*ERT2*^) displays accelerated onset while still recapitulating hallmark ALS features such as muscle atrophy and neuronal death, thereby constituting a valid and aggressive disease model. Compared with the classic ALS model [25, 27, 28], our model has distinct advantages: consistent pathology, no sex bias, and preserved viability and reproductive capacity prior to induction.

It is noteworthy that not all cell types with TDP‐43 knockout will exhibit ALS‐like phenotype. For example, TDP‐43 in astrocyte‐specific mice knockout causes only mild motor deficits without motor neuron degeneration [29]. Our study also found that after 8‐week tamoxifen induction, TDP‐43 in microglia‐specific knockout (*TDP‐43* ^*flox/flox*^; *Tmem119‐Cre* ^*ERT2*^) mice did not develop ALS‐like phenotypes such as motor dysfunction (unpublished, Figure S5). These findings reflect that neuronal TDP‐43 dysfunction is a critical determinant of ALS pathogenesis.

Glycolysis is essential for cerebral energy metabolism [30]. Neurons primarily rely on aerobic oxidation, but under conditions of mitochondrial dysfunction or tricarboxylic acid (TCA) cycle impairment, energy production shifts toward the less efficient anaerobic glycolysis. This compensatory shift requires increased glucose uptake and leads to excessive lactate accumulation [31, 32]. Consistent with this mechanism, studies in rotenone‐induced Parkinson's disease (PD) cell models have shown elevated expression and activity of the glycolytic key enzyme hexokinase‐2 (HK2), resulting in abnormally enhanced glycolytic flux and cellular injury [33]. Similarly, inhibiting glycolysis in microglia of AD model mice significantly ameliorates cognitive dysfunction [32]. In line with these findings, our study detected significantly elevated lactate levels in the motor cortex of TDP‐43 cKO mice. Furthermore, TDP‐43‐deficient NSC34 cells exhibited mitochondrial damage, increased glucose uptake, and lactate production, collectively confirming a pathological shift toward anaerobic glycolysis. Although anaerobic glycolysis can provide a short‐term energy supply, it also leads to a series of new problems.

While clinical data associate higher dietary glycemic load with prolonged survival in some ALS patients [34]—suggesting a beneficial compensatory role for glucose utilization during bioenergetic crisis—our findings reveal that TDP‐43 deficiency triggers excessive, ALDOA‐driven anaerobic glycolysis, creating a persistent high‐lactate environment. Although this glycolytic upregulation may initially serve as a compensatory response, it ultimately disrupts neuronal energy homeostasis, manifested as reduced ATP production, lactate accumulation, and acidosis, and may exacerbate neuronal damage via lactate‐mediated neuroinflammatory and oxidative stress signaling [35, 36].

As a key glycolytic enzyme, ALDOA critically regulates glycolytic efficiency through its activity and expression level, thereby modulating cellular energy metabolism and the underlying physiological functions [37]. Substantial evidence demonstrated ALDOA by promoting glycolytic flux and lactate production, thereby contributing to tumor progression [9]. Recent studies have shown that there is disrupted cerebral glucose metabolism in AD patients, and the expression levels of two glycolytic enzymes—pyruvate kinase (PKM) and aldolase A (ALDOA)—are significantly elevated in cerebrospinal fluid [38]. Currently, there is a lack of evidence regarding ALDOA in ALS patients. A study using a cell model expressing TDP‐43 mutations (A315T, M337V, and S379P) revealed that with prolonged expression of the TDP‐43 mutant protein, the levels of ALDOA and other glycolytic enzymes decrease in a time‐dependent manner [39]. In contrast, the present study demonstrates that TDP‐43 loss‐of‐function specifically upregulates ALDOA expression in the motor cortex, disrupting glucose metabolic homeostasis and driving neuronal degeneration. Therefore, the regulation of glycolytic enzymes by TDP‐43 pathology exhibits considerable complexity. This disparity underscores the divergent effects of TDP‐43 loss‐of‐function versus toxic gain‐of‐function on metabolic networks and suggests that glycolytic flux may be dynamically regulated across different stages or pathological conditions in ALS.

Aldometanib, a specific ALDOA inhibitor, blocks fructose‐1,6‐bisphosphate catalysis [19], inhibiting glycolysis and cellular energetics [40]. While its effects have been studied in oncology, its role in neurodegenerative diseases was previously unknown. This study demonstrated that aldometanib treatment significantly increased the number of surviving motor neurons in the motor cortex and extended the lifespan of TDP‐43 cKO mice. Although rotarod and hanging wire tests did not reveal statistically significant differences between groups, clear behavioral distinctions were identified at the terminal stage. On the 28th day after TM induction, untreated TDP‐43 cKO mice exhibited moribund status, whereas aldometanib‐treated mice were still able to perform spontaneous turning and slow crawling. Current approaches, such as lactate‐targeted therapies to support mitochondria [41] or those directly ameliorating mitochondrial dysfunction [42], primarily target downstream mechanisms and remain in preclinical exploration. In contrast, our study identifies ALDOA as a more upstream node within the pathogenic cascade, offering a new intervention target with potential therapeutic rationale for ALS.

Glycolysis is closely associated with pyroptosis [43]. ALDOA promotes NLRP3 inflammasome activation by sensing glycolytic flux, regulating AMPK activation, and inhibiting mitophagy [14]. Studies have shown that pyroptosis plays a key role in various neurodegenerative diseases [44, 45, 46, 47]. In ALS transgenic (*SOD1* ^*G93A*^) mice, activation of the canonical NLRP3 inflammasome can induce pyroptosis in ventral horn motor neurons, leading to motor neuron loss and neuroinflammation [48]. In our ALS model, we delineate a novel pathogenic mechanism whereby ALDOA drives neuronal pyroptosis by enhancing anaerobic glycolysis. Furthermore, this study provides the first assessment of aldometanib's neuroprotective effects, demonstrating its ability to inhibit anaerobic glycolysis and reduce neuronal degeneration and death during ALS pathogenesis. These results provide a new perspective for exploring the mechanism of motor neuron degeneration in ALS.

This study has several limitations. First, direct evidence for ALDOA in human ALS pathology is currently lacking. Although metabolic dysfunction is well documented in ALS [3, 4] and altered ALDOA expression in AD [38], its role in human ALS tissues or biofluids remains unexplored. Future studies should validate these findings using patient‐derived materials (post‐mortem tissue, biofluids, iPSC‐derived motor neurons). Second, technical limitations exist: enhanced glycolysis was not corroborated in diverse ALS models (including animal and iPSC‐derived), limiting generalizability; absence of ALDOA knockdown models precludes precise evaluation of its direct role in disease progression and target specificity; aldometanib's pharmacokinetics, blood–brain barrier penetration, and selectivity for ALDOA over other aldolase isoforms remain to be thoroughly characterized. These limitations will be systematically addressed through broader disease models, human tissue validation, and in‐depth mechanistic and pharmacological studies.

## 4 Conclusions

In conclusion, this study demonstrated that ALDOA‐mediated glycolytic enhancement could exacerbate neuroinflammation, induce motor neuron pyroptosis, and accelerate ALS progression. Conversely, glycolysis inhibition suppressed the NLRP3/GSDMD pathway, reduced pyroptotic neuronal death, and delayed disease progression. These findings identify ALDOA as a critical modulator of metabolic–inflammatory crosstalk and a promising therapeutic target for ALS. This research deepened the understanding of ALS pathogenesis and provided a theoretical basis for the development of novel metabolic‐based treatment strategies.

## Author Contributions

F.B. and Y.L. contributed to the conception and design of the study; T.Z. and X.G. contributed to the acquisition and analysis of data; T.Z. and K.Y. contributed to drafting the text and preparing figures.

## Funding

This study was supported by the National Natural Science Foundation of China (Grant Nos. 82171433 and 81760238), the Natural Science Foundation of Hunan Province of China (Grant Nos. 2022JJ30918 and 2023JJ30927), the Xinjiang Uygur Autonomous Region Health Commission (“Tianshan Talent” High‐Level Medical and Health Personnel Project, Grant No. TSYC202401B119), and the Natural Science Foundation of Xinjiang Uygur Autonomous Region (Grant No. 2025D01E38).

## Conflicts of Interest

The authors declare no conflicts of interest.

## Supporting information


**Figure S1:** Validation of TDP‐43 cKO mice. (A) Schematic diagram of the mouse breeding strategy. (B, C) Representative Western blot images and corresponding quantification of TDP‐43 protein levels in the motor cortex of each group of mice. (D) Statistical analysis of TDP‐43 mRNA levels in the motor cortex of each group. (E) Immunofluorescence co‐staining for Map2 and TDP‐43 in the motor cortex of each group. Data are presented as Mean ± SD. Statistical analyses in (C) and (D) were performed using the *t*‐test. *p < 0.05, ****p < 0.001 vs. con group.

**Figure S2:** Validation of TDP‐43 knockdown in NSC34 cells. (A) Representative Western blot image following TDP‐43 knockdown. (B) Quantification of TDP‐43 protein levels shown in (A). (C) Statistical analysis of TDP‐43 mRNA levels in each group of cells. Data are presented as Mean ± SD. Statistical analyses in (B) and (C) were performed using a two‐tailed unpaired *t*‐test. *p < 0.05, ****p < 0.0001 vs. con group.

**Figure S3:** 1 nM Aldometanib ameliorates the TDP‐43 knockdown‐induced decline in cell viability and activation of the NLRP3 inflammasome. (A) and (B) show the representative Western blot bands and the statistical results of NLRP3 protein levels in each group of cells, respectively; (C) presents the statistical graph of cell viability across groups. Data are presented as Mean ± SD. Statistical analyses in (B) and (C) were performed using one‐way analysis of variance (one‐way ANOVA). *p < 0.05, **p < 0.01, ***p < 0.001, ****p < 0.0001.

**Figure S4:** Increased microglial activation in the motor cortex of TDP‐43 cKO mice. Representative images of Iba1 immunofluorescence staining in the motor cortex of each group of mice. Scale bar: 20 μm in the left panel; 50 μm in the right magnified panel.

**Figure S5:** acn370372‐sup‐0005‐FigureS5.docx. *TDP‐43* ^*flox/flox*^ *; Tmem119‐2A‐Cre* ^*ERT2*^mice were successfully generated but did not exhibit ALS‐like phenotypes. (A, B) Statistical graphs of rotarod test and wire hanging test results for each group, respectively. (C) Statistical graph of body weight changes in each group. (D) Comparative images of body sizes in each group 56 days after tamoxifen injection. (E) Representative images of Nissl staining in the motor cortex of each group. (F) Quantitative analysis of Nissl‐positive neurons. Scale bars: 20 μm (left panel of E), 50 μm for magnified view (right panel of E). Data are presented as mean ± SD. A–C: Two‐way ANOVA; ns (not significant) indicates *p* > 0.05 versus *TDP‐43* ^*flox/flox*^ group.

**Table S1:** Primers used for RT‐qPCR.

## Data Availability

The data that support the findings of this study are available within the article and its Supporting Information.
