# Supplementary material for: ALDOA Promotes Glycolysis and NLRP3/GSDMD Pyroptosis to Accelerate ALS Progression
Source: Ann Clin Transl Neurol. 2026 Mar 24;13(9):1903–18. doi: 10.1002/acn3.70372 (PMC13394068; doi:10.1002/acn3.70372)
Supplement: Supplementary file 2 [file ACN3-13-1903-s002.docx]

Figure S1: Validation of TDP-43 cKO mice. (A) Schematic diagram of the mouse breeding strategy. (B, C) Representative Western blot images and corresponding quantification of TDP-43 protein levels in the motor cortex of each group of mice. (D) Statistical analysis of TDP-43 mRNA levels in the motor cortex of each group. (E) Immunofluorescence co-staining for Map2 and TDP-43 in the motor cortex of each group. Data are presented as Mean ± SD. Statistical analyses in (C) and (D) were performed using the t-test. *p < 0.05, ****p < 0.001 vs con group.
