# Supplementary material for: ALDOA Promotes Glycolysis and NLRP3/GSDMD Pyroptosis to Accelerate ALS Progression
Source: Ann Clin Transl Neurol. 2026 Mar 24;13(9):1903–18. doi: 10.1002/acn3.70372 (PMC13394068; doi:10.1002/acn3.70372)
Supplement: Supplementary file 4 [file ACN3-13-1903-s004.docx]

Figure S2. Validation of TDP-43 knockdown in NSC34 cells. (A) Representative Western blot image following TDP-43 knockdown. (B) Quantification of TDP-43 protein levels shown in (A). (C) Statistical analysis of TDP-43 mRNA levels in each group of cells. Data are presented as Mean ± SD. Statistical analyses in (B) and (C) were performed using a two-tailed unpaired t-test. *p < 0.05, ****p < 0.0001 vs con group.
