# Supplementary material for: ALDOA Promotes Glycolysis and NLRP3/GSDMD Pyroptosis to Accelerate ALS Progression
Source: Ann Clin Transl Neurol. 2026 Mar 24;13(9):1903–18. doi: 10.1002/acn3.70372 (PMC13394068; doi:10.1002/acn3.70372)
Supplement: Supplementary file 5 [file ACN3-13-1903-s005.docx]

**Table S1. Primers used for RT-qPCR**

| **Name** | **Sequences (5’-3’)** |
| --- | --- |
| TDP43-FORWARD | TACCCTTACCTTCACCTCGTC |
| TDP43-REVERSE | GAUGGGAAAAGAAG-GUGGCGACCUUCCC |
| ALDOA-FORWARD | GGAACCAATGGCGAGACAACTACC |
| ALDOA-REVERSE | GGCAAAGTCGGCTCCATCCTTC |
| β-actin-FORWARD | GGTTCCGCTGCCCTGAGG |
| β-actin-REVERSE | GGAGTTGAAGGTAGTTTCGTGGATG |
