# Supplementary material for: ALDOA Promotes Glycolysis and NLRP3/GSDMD Pyroptosis to Accelerate ALS Progression
Source: Ann Clin Transl Neurol. 2026 Mar 24;13(9):1903–18. doi: 10.1002/acn3.70372 (PMC13394068; doi:10.1002/acn3.70372)
Supplement: Supplementary file 6 [file ACN3-13-1903-s006.docx]

Figure S4:Increased microglial activation in the motor cortex of TDP-43 cKO mice. Representative images of Iba1 immunofluorescence staining in the motor cortex of each group of mice. Scale bar: 20 μm in the left panel; 50 μm in the right magnified panel.
