# Supplementary material for: ALDOA Promotes Glycolysis and NLRP3/GSDMD Pyroptosis to Accelerate ALS Progression
Source: Ann Clin Transl Neurol. 2026 Mar 24:10.1002/acn3.70372. Online ahead of print. doi: 10.1002/acn3.70372 (PMC13394068; doi:10.1002/acn3.70372)
Supplement: Supplementary file 1 — Figure S1: Validation of TDP‐43 cKO mice. (A) Schematic diagram of the mouse breeding strategy. (B, C) Representative Western blot images and corresponding quantification of TDP‐43 protein levels in the motor cortex of each group of mice. (D) Statistical analysis of TDP‐43 mRNA levels in the motor cortex of each group. (E) Immunofluorescence co‐staining for Map2 and TDP‐43 in the motor cortex of each group. Data are presented as Mean ± SD. Statistical analyses in (C) and (D) were performed using the t‐test. *p < 0.05, ****p < 0.001 vs. con group. [file ACN3-9999-0-s001.docx]

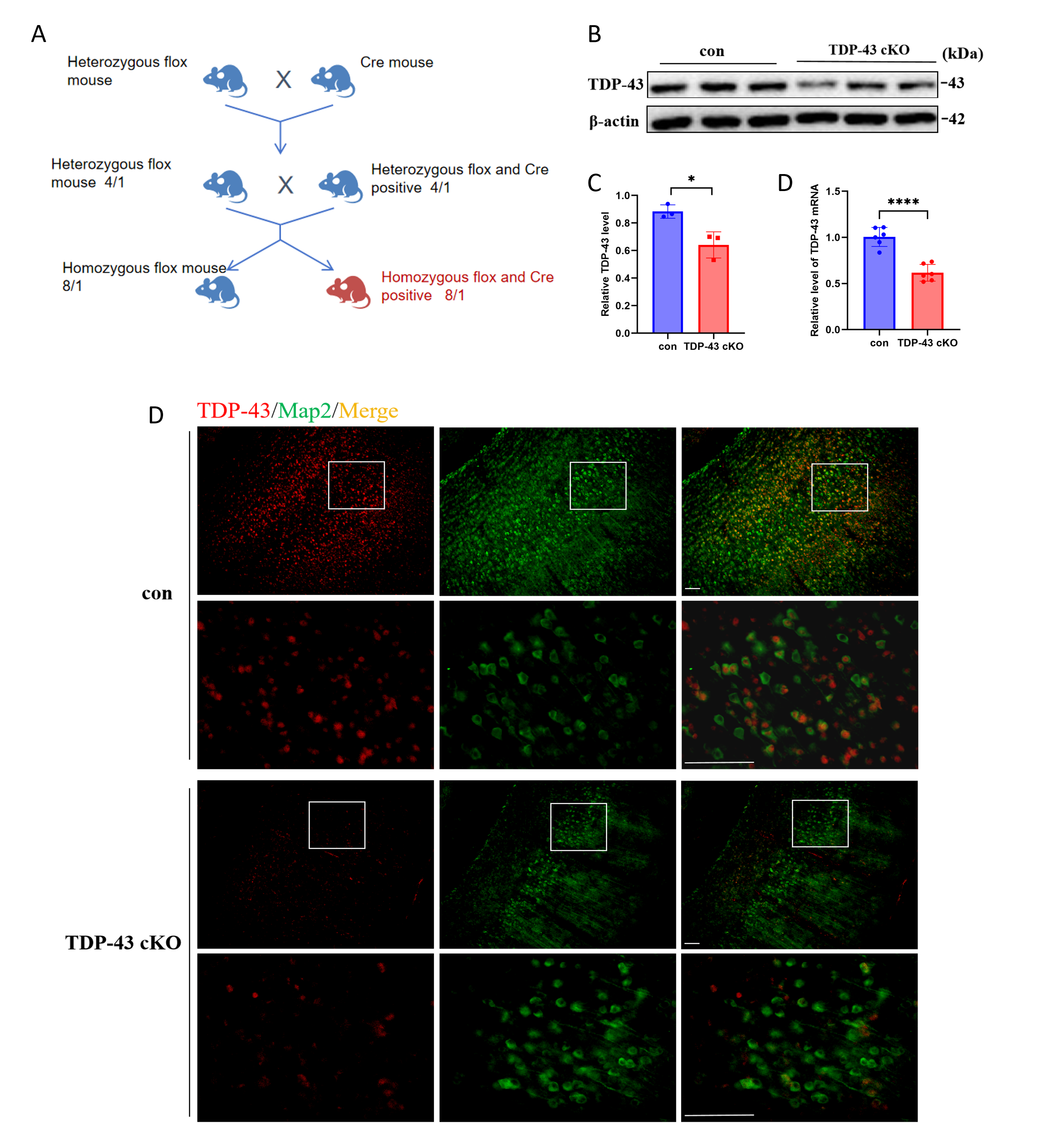


Figure S1: Validation of TDP-43 cKO mice. (A) Schematic diagram of the mouse breeding strategy. (B, C) Representative Western blot images and corresponding quantification of TDP-43 protein levels in the motor cortex of each group of mice. (D) Statistical analysis of TDP-43 mRNA levels in the motor cortex of each group. (E) Immunofluorescence co-staining for Map2 and TDP-43 in the motor cortex of each group. Data are presented as Mean ± SD. Statistical analyses in (C) and (D) were performed using the t-test. *p < 0.05, ****p < 0.001 vs con group.
