# Supplementary material for: ALDOA Promotes Glycolysis and NLRP3/GSDMD Pyroptosis to Accelerate ALS Progression
Source: Ann Clin Transl Neurol. 2026 Mar 24:10.1002/acn3.70372. Online ahead of print. doi: 10.1002/acn3.70372 (PMC13394068; doi:10.1002/acn3.70372)
Supplement: Supplementary file 2 — Figure S2: Validation of TDP‐43 knockdown in NSC34 cells. (A) Representative Western blot image following TDP‐43 knockdown. (B) Quantification of TDP‐43 protein levels shown in (A). (C) Statistical analysis of TDP‐43 mRNA levels in each group of cells. Data are presented as Mean ± SD. Statistical analyses in (B) and (C) were performed using a two‐tailed unpaired t‐test. *p < 0.05, ****p < 0.0001 vs. con group. [file ACN3-9999-0-s004.docx]

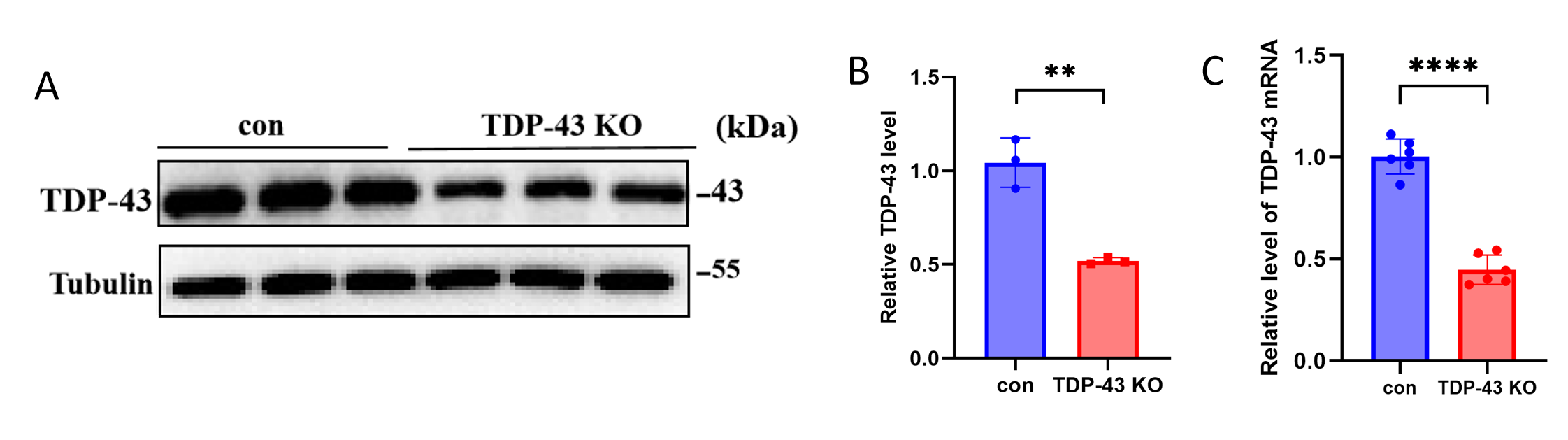
Figure S2. Validation of TDP-43 knockdown in NSC34 cells. (A) Representative Western blot image following TDP-43 knockdown. (B) Quantification of TDP-43 protein levels shown in (A). (C) Statistical analysis of TDP-43 mRNA levels in each group of cells. Data are presented as Mean ± SD. Statistical analyses in (B) and (C) were performed using a two-tailed unpaired t-test. *p < 0.05, ****p < 0.0001 vs con group.
