# Supplementary material for: ALDOA Promotes Glycolysis and NLRP3/GSDMD Pyroptosis to Accelerate ALS Progression
Source: Ann Clin Transl Neurol. 2026 Mar 24:10.1002/acn3.70372. Online ahead of print. doi: 10.1002/acn3.70372 (PMC13394068; doi:10.1002/acn3.70372)
Supplement: Supplementary file 3 — Figure S3: 1 nM Aldometanib ameliorates the TDP‐43 knockdown‐induced decline in cell viability and activation of the NLRP3 inflammasome. (A) and (B) show the representative Western blot bands and the statistical results of NLRP3 protein levels in each group of cells, respectively; (C) presents the statistical graph of cell viability across groups. Data are presented as Mean ± SD. Statistical analyses in (B) and (C) were performed using one‐way analysis of variance (one‐way ANOVA). *p < 0.05, **p < 0.01, ***p < 0.001, ****p < 0.0001. [file ACN3-9999-0-s005.docx]

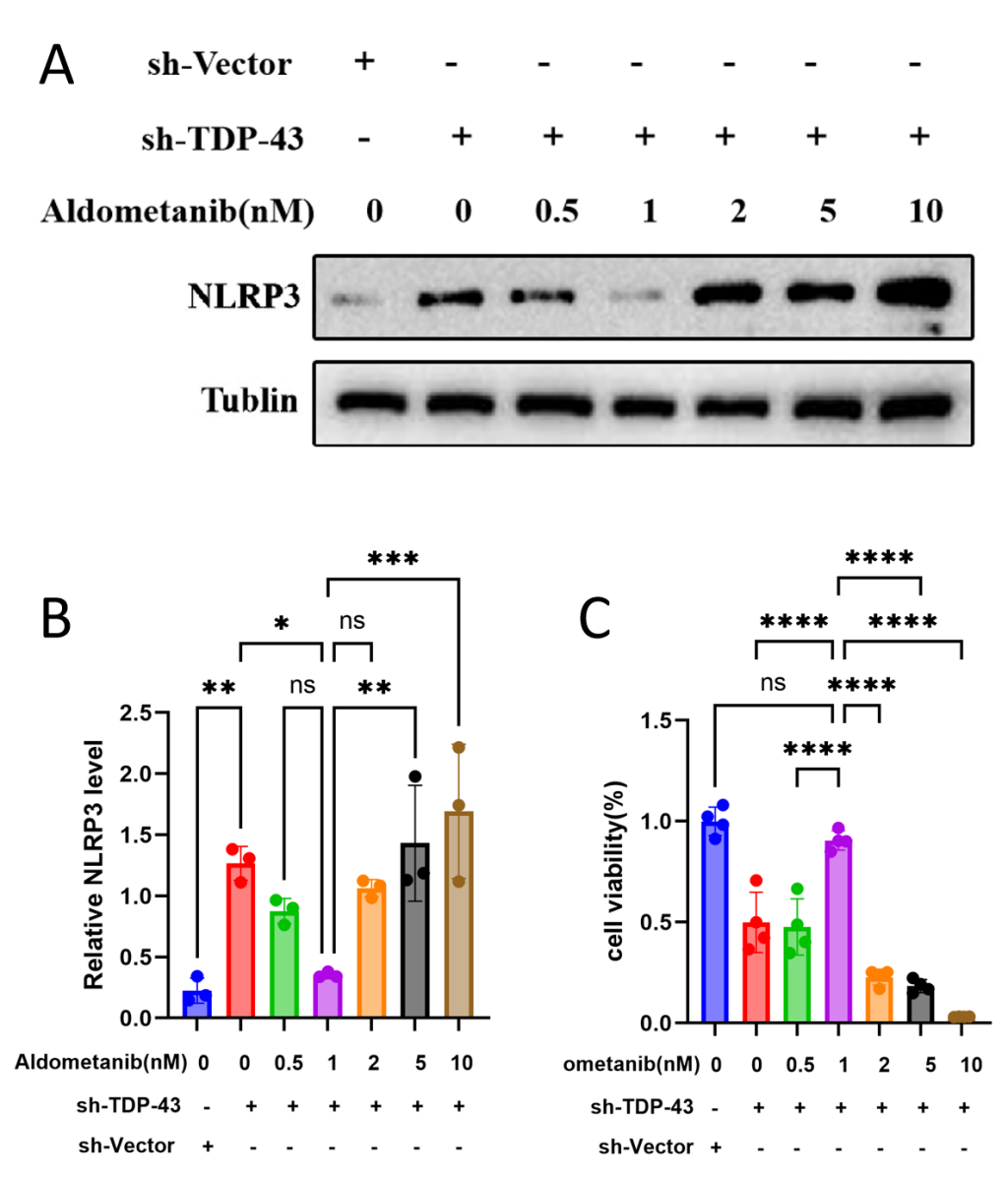


Figure S3. 1 nM Aldometanib ameliorates the TDP-43 knockdown-induced decline in cell viability and activation of the NLRP3 inflammasome. (A) and (B) show the representative Western blot bands and the statistical results of NLRP3 protein levels in each group of cells, respectively; (C) presents the statistical graph of cell viability across groups.Data are presented as Mean ± SD. Statistical analyses in (B) and (C) were performed using One-way analysis of variance (one-way ANOVA). *p < 0.05, **p < 0.01, ***p < 0.001 ,****p < 0.0001 .
