# Supplementary material for: ALDOA Promotes Glycolysis and NLRP3/GSDMD Pyroptosis to Accelerate ALS Progression
Source: Ann Clin Transl Neurol. 2026 Mar 24:10.1002/acn3.70372. Online ahead of print. doi: 10.1002/acn3.70372 (PMC13394068; doi:10.1002/acn3.70372)
Supplement: Supplementary file 4 — Figure S4: Increased microglial activation in the motor cortex of TDP‐43 cKO mice. Representative images of Iba1 immunofluorescence staining in the motor cortex of each group of mice. Scale bar: 20 μm in the left panel; 50 μm in the right magnified panel. [file ACN3-9999-0-s002.docx]

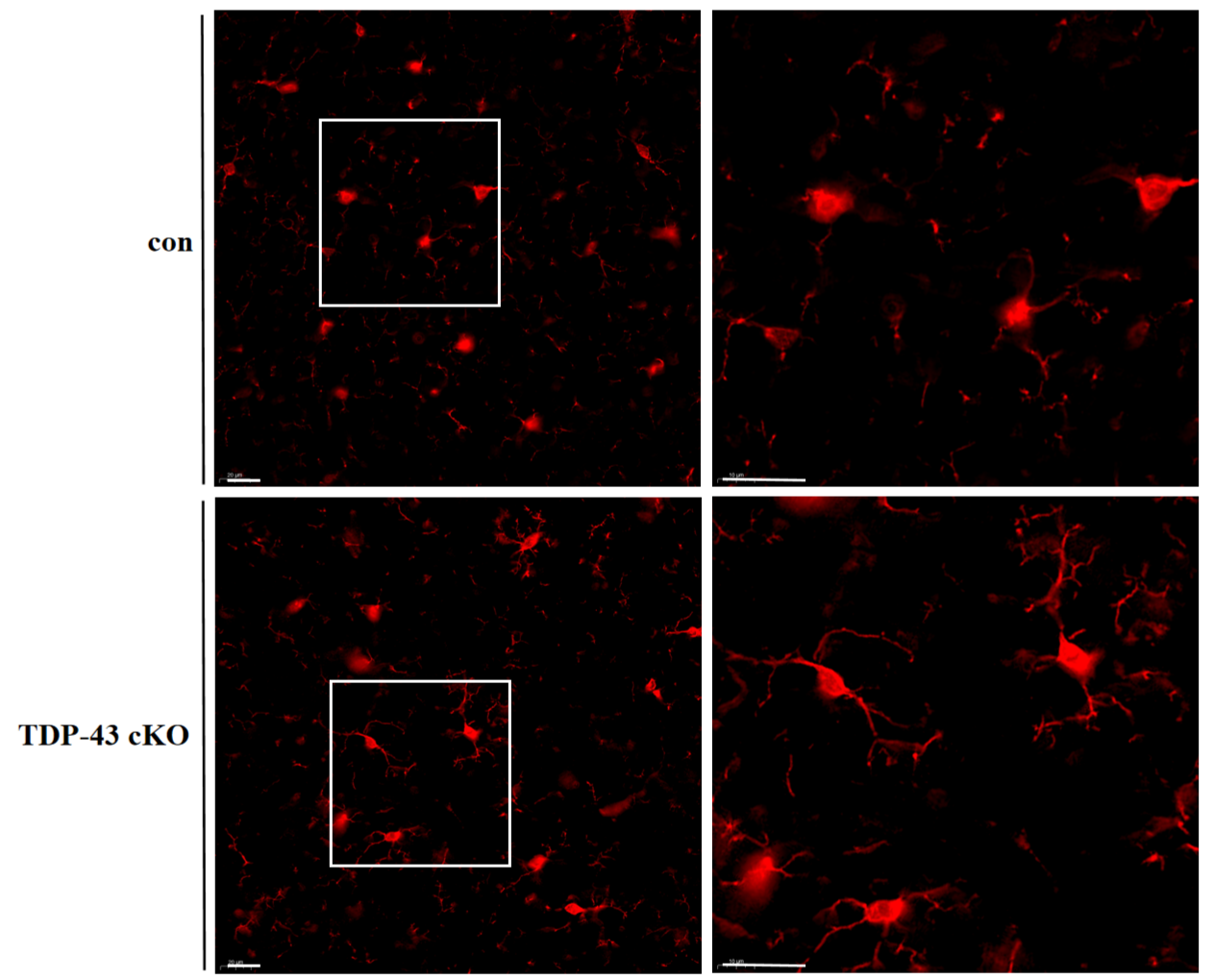


Figure S4:Increased microglial activation in the motor cortex of TDP-43 cKO mice. Representative images of Iba1 immunofluorescence staining in the motor cortex of each group of mice. Scale bar: 20 μm in the left panel; 50 μm in the right magnified panel.
