# Supplementary material for: ALDOA Promotes Glycolysis and NLRP3/GSDMD Pyroptosis to Accelerate ALS Progression
Source: Ann Clin Transl Neurol. 2026 Mar 24:10.1002/acn3.70372. Online ahead of print. doi: 10.1002/acn3.70372 (PMC13394068; doi:10.1002/acn3.70372)
Supplement: Supplementary file 5 — Figure S5: acn370372‐sup‐0005‐FigureS5.docx. TDP‐43 flox/flox ; Tmem119‐2A‐Cre ERT2 mice were successfully generated but did not exhibit ALS‐like phenotypes. (A, B) Statistical graphs of rotarod test and wire hanging test results for each group, respectively. (C) Statistical graph of body weight changes in each group. (D) Comparative images of body sizes in each group 56 days after tamoxifen injection. (E) Representative images of Nissl staining in the motor cortex of each group. (F) Quantitative analysis of Nissl‐positive neurons. Scale bars: 20 μm (left panel of E), 50 μm for magnified view (right panel of E). Data are presented as mean ± SD. A–C: Two‐way ANOVA; ns (not significant) indicates p > 0.05 versus TDP‐43 flox/flox group. [file ACN3-9999-0-s006.docx]

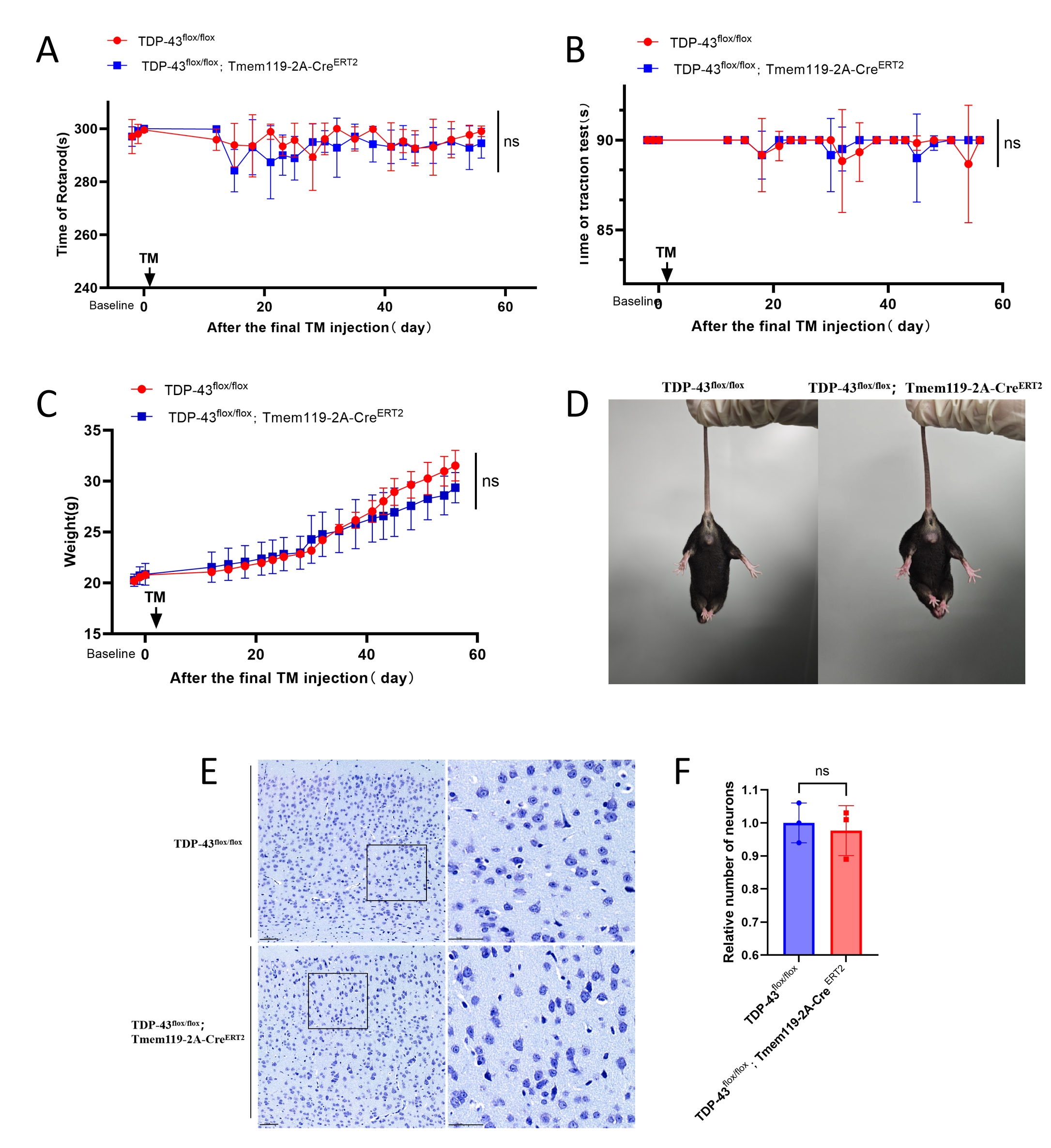


Figure S5: *TDP-43^flox/flox^; Tmem119-2A-Cre^ERT2^*mice were successfully generated but did not exhibit ALS-like phenotypes. (A, B)Statistical graphs of rotarod test and wire hanging test results for each group, respectively. (C) Statistical graph of body weight changes in each group. (D) Comparative images of body sizes in each group 56 days after tamoxifen injection. (E) Representative images of Nissl staining in the motor cortex of each group. (F) Quantitative analysis of Nissl-positive neurons. Scale bars: 20 μm (left panel of E), 50 μm for magnified view (right panel of E).Data are presented as mean ± SD. A, B, C: Two-way ANOVA; ns (not significant) indicates *P* > 0.05 versus *TDP-43^flox/flox^* group.
