# Supplementary material for: ALDOA Promotes Glycolysis and NLRP3/GSDMD Pyroptosis to Accelerate ALS Progression
Source: Ann Clin Transl Neurol. 2026 Mar 24:10.1002/acn3.70372. Online ahead of print. doi: 10.1002/acn3.70372 (PMC13394068; doi:10.1002/acn3.70372)
Supplement: Supplementary file 6 — Table S1: Primers used for RT‐qPCR. [file ACN3-9999-0-s003.docx]

**Table S1. Primers used for RT-qPCR**

| **Name** | **Sequences (5’-3’)** |
| --- | --- |
| TDP43-FORWARD | TACCCTTACCTTCACCTCGTC |
| TDP43-REVERSE | GAUGGGAAAAGAAG-GUGGCGACCUUCCC |
| ALDOA-FORWARD | GGAACCAATGGCGAGACAACTACC |
| ALDOA-REVERSE | GGCAAAGTCGGCTCCATCCTTC |
| β-actin-FORWARD | GGTTCCGCTGCCCTGAGG |
| β-actin-REVERSE | GGAGTTGAAGGTAGTTTCGTGGATG |
